# A Catalogue of Altered Salivary Proteins Secondary to Invasive Ductal Carcinoma: A Novel *In Vivo* Paradigm to Assess Breast Cancer Progression

**DOI:** 10.1038/srep30800

**Published:** 2016-08-01

**Authors:** Charles F. Streckfus, Lenora Bigler

**Affiliations:** 1 University of Texas School of Dentistry at Houston Department of Diagnostic and Behavioral Sciences Behavioral & Biomedical Sciences Building, Rm. 5322 Houston, Texas 77054, USA.

## Abstract

The objective of this manuscript is to introduce a catalogue of salivary proteins that are altered secondary to carcinoma of the breast. The catalogue of salivary proteins is a compilation of twenty years of research by the authors and consists of 233 high and low abundant proteins which have been identified by LC-MS/MS mass spectrometry, 2D-gel analysis and by enzyme-linked immunosorbent assay. The body of research suggests that saliva is a fluid suffused with solubilized by-products of oncogenic expression and that these proteins may be useful in the study of breast cancer progress, treatment efficacy and the tailoring of individualized patient care.

Despite the numerous advances made in breast cancer research, carcinoma of the breast is still the most common, disfiguring and deadliest cancer among women^1^. Screening for breast cancer has led to earlier detection; however, the age old dilemma of “watch-and-wait” or to aggressively treat breast lesions haunt clinicians^1^,^2^. The understanding of BRCA1 and BRCA2 gene mutations has saved lives, but these findings account for only 5 to 10% of breast cancer cases^1^. Additional paradigms, i.e. biological models, may be needed in order to increase early detection and treatment efficacy^1^,^2^.

Biological models, such as those for breast cancer, simulate the simultaneous operations and interactions of multiple processes and molecular networks, in an attempt to re-create and predict the appearance of complex phenomena such as breast cancer progression^3^. A model that can reflect the presence and progression of malignancy among women would enhance our knowledge of breast cancer, and serve as an enabling system for biomarker discovery and credentialing of candidate markers so that the biological causality of any analyte(s) can be assessed. With respect to the study of breast cancer, there are currently three major methods for creating models for studying breast cancer progression. The three methods utilize either breast cancer tumor cell lines (BCTCL), xenografts of cell lines, and the third method uses animals – in this case genetically engineered mice for creating various models for studying breast cancer^1^. All three models have generated useful insight into cancer progression; however, despite their utility no individual model recapitulates all aspects of cancer progression^4^.

In the case of BCTCL research, the main question is - are the cell lines representative of human breast cancer and how well do they capture breast cancer tumorigenesis in the context of the unique tumor-stroma microenvironment? This question is debatable with strong evidence supporting both sides of the discussion. The fact is that no single cell line is representative of the disease process. To date, there are 51 BCTCL used to detect genetic abnormalities associated with breast cancer. However, the major concern is that multiple variants of the same cell line may exist and display distinct phenotypes. This suggests that they have acquired genetic changes that might preclude comparison between different studies^1^,^2^,^3^. Moreover, the ability of *ex vivo* cell line experiments to recapitulate the tumor microenvironment is in question, with stoma and extracellular matrix interactions, immune cell involvement, vasculature, and the complex milieu of the blood itself all contributing to the tumor cell biology. Additionally, cell lines are prone to genotypic and phenotypic drift during their continual culture and there is always the problem of contamination^1^,^2^,^3^,^4^.

Xenografts models are also very useful in studying breast cancer and have certainly helped understand genetic pathways associated with breast cancers but they are also problematic. Many of the problems that plague cell line studies also affect xenograft-driven modeling; however, a major problem is that xenografts must be established in immunocompromised mice^4^,^5^,^6^. The absence of an intact immune system may profoundly affect tumor development and progression as this microenvironment is entirely artificial. In fact, there is increasing evidence of roles for the immune system in both early stage breast cancer and metastasis^1^,^4^,^5^,^6^,^7^. There are also stoma differences between mouse and human breast tissues the must be taken into account. However, the major problem with xenograft models has to deal with breast cancer metastasis because metastatic cells preferentially colonize the lungs in mice, (instead of developing brain and liver metastasis) contain less fibrosis and inflammation then human tumors, nearly all are hormone independent as opposed to approximately half of human breast cancers that are hormone dependent, Another major draw-back is the lack of histological concordance between tumors from genetically engineered mice and the common types of human breast cancers. Finally, most tumors from mice do not resemble the most common subtypes of breast cancer. Taken together, clinicians and researchers may benefit by having an additional model system for assessing breast cancer tumorigenesis and in particular a predictive model for treatment response^4^.

One question emanating from the aforementioned paragraphs is what is the alternative to those models? The answer we are proposing is the use of the salivary proteome to study and assess cancer progression. The basic secretory units of the salivary glands are phenotypically similar to those of the mammary glands^8^,^9^,^10^,^11^,^12^. With the submandibular gland being the exception; they both have origins from the ectodermal germ layer. Both tissues are compound exocrine glands composed of specialized glandular epithelia. The tissues are characterized with two epithelial cell types i.e., ductal and acinar cells along with myoepithelial cells which contract to move fluid from the acinar lumen to the ducts. The ductal epithelial cells (terminal ducts) adjacent to the acinar units are cuboidal^8^,^9^,^10^,^11^,^12^.

From an immunohistological perspective there are a number of similarities between the mammary and salivary gland tissues. Both tissues have HER2/neu receptors on their ductal epithelial cells which can be overexpressed in malignant transformation^8^,^9^,^10^,^11^,^12^. Additionally, epithelial cells of both tissues have estrogen, progesterone, and androgen receptors that can be overexpressed^8^,^9^,^10^,^11^,^12^. p53 tumor suppressor gene is often mutated with overexpression of ineffective, mutated protein in carcinomas of the breast or the salivary glands. More interesting is that these proteins can be found in mammary ductal fluids and saliva^8^,^9^,^10^,^11^,^12^. As a consequence, the purpose of this manuscript is to provide evidence for the use of salivary protein profiles as an *in vivo* adjunct model for studying breast cancer progression.

## Results and Discussions

The results of the proteomic analysis yielded 233 proteins that were either up or down regulated secondary to the presence of carcinoma of the breast. The 233 proteins represent 46% of the total number of proteins (505) identified among normal individuals assayed in this report. Table 1 lists the 233 proteins. Of the 233 proteins, 142 were up-regulated and 91 were down regulated. The down regulated proteins are emboldened in Table 1. In addition, the profile consists of both high and low abundant salivary proteins assayed by both mass spectrometry and enzyme-linked immunosorbent assay (ELISA) respectively.

**Figure 1:**
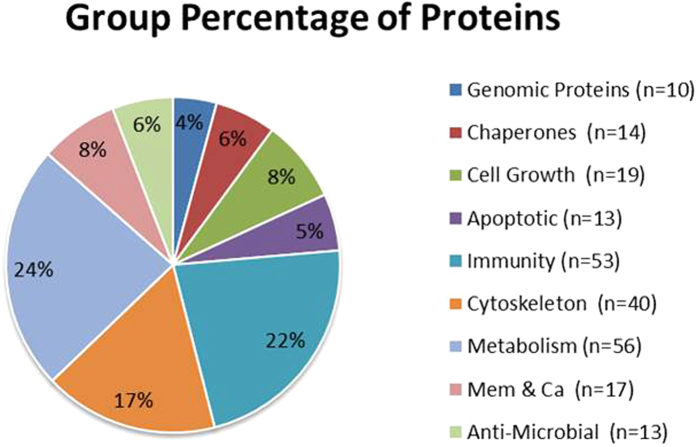
The figure represents the number and percentage of proteins as classified by function.

In order to compare the results to published proteomic cancer cell analysis, the proteins were categorized into ten groups of cellular activity^13^,^14^. The groups are illustrated in Fig. 1 and are as follows: 1) Genomic proteins; 2) molecular chaperones; 3) cell growth; 4) apoptotic proteins; 5) anti-inflammatory and immunoresponse proteins; 6) cytoskeletal proteins; 7) metabolic proteins; 8) membrane associated proteins and 9) antimicrobial proteins^13^,^14^.

As illustrated in Fig. 1 and Table 1, the metabolic protein category reflected 24% of the 236 proteins while the inflammatory/immunoresponse and the cytoskeletal categories exhibited 23% and 17% of the total number of proteins respectively. Eighty four (36%) of the proteins were detected in the early stage carcinomas (Stage 0 & Stage I).

Table 2 demonstrates how each protein was assayed and which proteins were present in varying cancer cell lines cited in the literature^15^,^16^,^17^,^18^. Fifty one of the 233 proteins (22%) could be identified in the SKBR3 cell line, 34 (14%) in the MCF7, 4 (2%) in the T47D, 29 (12%) in the MB-MDA-231, 26 (11%) in the 8701-BC and 43 (18%) in malignant tumor tissues. Twenty four (11%) were common to three or more of the cell lines.

Additionally, information is provided as to which proteins are contain within both salivary and breast tissue exosomes. Seventy one (30%) proteins from the panel were found to be contained within salivary exosomes and 35 (15%) proteins were within breast cancer tissue exosomes. There were twenty seven (11%) proteins that were common to both the salivary and the breast tissue exosomes.

Table 3 displays the results from the GO and AmiGO analyses. It illustrates the overlapping functional diversity of the panel with respect to their associated molecular processes.

Table 4 represents a sampling of some of the significant pathways calculated by the National Cancer Institute’s Pathway Interaction Database. The signaling events mediated by HDAC Class III was highly significant at the p < 0.01 × 10^−16^ level as was the glucocorticoid receptor regulatory network at the p < 0.01 × 10^−6^ level.

The analyses for nodal & Her2/neu receptor status were also performed and published prior to this manuscript^10^,^11^. Briefly, the results yielded approximately 174 differentially expressed proteins in the saliva specimens lymph node status. There were 55 proteins that were common to both cancer stages in comparison to each other and healthy controls. In contrast, there were there were 20 proteins unique to Stage IIa and 28 proteins that were unique to Stage IIb^10^. The results Her2/neu receptor status yielded approximately 71 differentially expressed proteins in the saliva specimens. There were 34 up-regulated proteins and 37 down regulated proteins^11^.

The results of the Harvard Partners Center for Genetics and Genomics, Cambridge, MA., proteomic analyses confirmed our findings and provided us with additional markers for this manuscript^12^.

The ensuing paragraphs further detail each protein category and how the proteins of each category relate to *carcinogenesis of the breast*.

### Genomic Integrity Related Proteins (10, 4%)

Ten salivary proteins related to genomic integrity were found to be variant (over-expressed) in the presence of carcinoma of the breast. Eight of the ten proteins were histones while the remaining two proteins were associated with genomic maintenance. Of the two aforementioned proteins, the TLS oncogene maintains genomic integrity and mRNA/microRNA processing, while the other protein, Proliferating Cell Nuclear Antigen, is implicated with DNA repair. Both proteins are involved in breast cancer progression^19^,^20^.

The remaining eight belong to the histone family of proteins. Histones are a group of basic proteins that are involved with nuclear DNA and help condense it into chromatin^21^. Histones are basic proteins, and their positive charges allow them to associate with DNA, which is negatively charged. Some histones function as molecular reels for the thread-like DNA to wrap around. Each histone octamer is composed of two copies each of the histone proteins H2A, H2B, H3, and H4. The chain of nucleosomes is then wrapped into a 30 nm spiral called a solenoid, where additional H1 histone proteins are associated with each nucleosome to maintain the chromosome structure^19^,^20^,^21^,^22^,^23^,^24^,^25^.

Of particular interest within the group of histones is the H2A family. This group in particular is epigenetically associated with carcinoma of the breast^26^. The H2AX variant for example (Table 1), functions as a sensor of DNA damage and responds by defining the cellular response for DNA repair or apoptosis. It is also an indicator of tumor radio-sensitivity and is associated with BRCA1 and E-Cahedrin1 activity. E-Cahedrin1 was found to be down regulated in saliva^25^,^26^,^27^.

Histone 3.2 is also present in saliva in the protein profile and is associated with invasive ductal cell carcinoma^28^. It is routinely used in tumor cell immunohistochemistry to determine the grade of the tumor. The presence of the over-expressed H3, H2AX and p300 proteins may possibly explain the upregulated presence of p21^(Waf−1)^ and CA 15-3 in saliva^29^.

### Molecular Chaperones/Heat Shock proteins (14, 6%)

Heat shock proteins (HSPs), also referred to as stress proteins, are a group of cellular proteins that respond to extreme temperature changes, infection, inflammation and oxygen deprivation. The proteins function as molecular chaperones and assist in the folding and maintenance of newly translated proteins, the refolding of denatured proteins and the further unfolding of misfolded or destabilized proteins to assist in their degradation^30^. Alterations in HSP expression may also increase due to other sources of cellular stress, including osmotic stress and the unfolded protein response, mediated by the ATF family of transcription factors. HSP expression and function can be deregulated during pathophysiological processes such as breast carcinogenesis^30^.

Table 1 exhibit fourteen proteins that function as molecular chaperones. Included among these are the overexpressed proteins Hsp27, Hsp40 and Hsp70. Hsp70 is especially important as this chaperone can block the programmed cell death that often accompanies malignant transformation. Additionally, Hsp70 may be involved in mitotic spindle formation and cell proliferation^31^.

In addition to the heat shock proteins, there were a number of proteins associated with protein folding and the catalyzation of –S–S– bonds. Of importance is the protein disulfide isomerase (PDI), which has shown to be upregulated in breast cancer tissues^30^,^31^.

### Growth Factors and Their Receptors (19, 7%)

Growth factors are polypeptides that stimulate cell proliferation by binding to membrane receptors; EGF for example has been cited as a salivary protein for nearly four decades and is recognized as being up-regulated in the presence of carcinoma of the breast along with the solubilized receptors EGFR1 and Her2/neu^32^,^33^. TGFα, another ligand of the EGF/Her2 signaling pathway, was found to over-expressed. Within the EGF/Her2 signaling pathway, the following downstream proteins were overexpressed: AKT1, CALM, CDH1, CALM3, EGF, EGFR, GRB2, MUC1, PAX, RAC1, STAT1, and UB^34^.

VEGF, a growth factor implicated in angiogenesis was also upregulated as were its associated downstream proteins AKT1, FGF2, FYN, NOS1, NOS3, p21, TNFα and VAV3^35^.

### Apoptotic Related Proteins (13, 5%)

There were thirteen proteins related to apoptosis that are presented in Table 1. Among the thirteen proteins there were five prominent proteins of the intrinsic apoptotic pathway: p53, Apaf-1, 14-3-3δ, Xiap and p21^WAF−1^. p53 and Apaf-1 were down regulated while14-3-3δ, Xiap and p21^WAF−1^ were up-regulated. This pattern appears to be consistent with cancer progression as cellular proliferation is one the hallmarks of this disease^36^. For example XIAP is a protein that impedes apoptotic cell death. XIAP is a member of the inhibitor of apoptosis family of proteins and is the most potent human IAP protein currently identified. In addition, there is the up-regulation of 14-3-3δ among this group of proteins. 14-3-3δ is a p53 inhibitor via Mdm2 inactivation. Additionally, when bound to KRT17, it regulates protein synthesis and cell growth by stimulating the Akt/mTOR pathway^36^,^37^,^38^.

Finally, the up-regulated presence of the p21^WAF−1^ protein suggests a possible anti-apoptotic role by suppressing pro-apoptotic genes. Phosphorylated p21^WAF1^ expression, for example in breast cancer, may be associated with an inability of p21^WAF1^ to inhibit cell cycle progression^38^,^39^,^40^. Additionally, p21^WAF1^, when phosphorylated via PI3K pathway may bind with PCNA and facilitate the inactivation of capase-3 which is essential in the apoptotic process. It can also bind with PCNA inhibiting DNA repair. The p21^WAF1^ may also be unable to regulate Cdc25C binding with PCNA which is necessary for cell cycle arrest the G_2_/M checkpoint^41^,^42^. Collectively, the aforementioned panel of proteins may have utility in assessing anti-apoptotic activity in cancer progression^36^,^37^,^38^,^39^,^40^,^41^,^42^,^43^.

### Chronic Inflammatory/Immunoresponse Proteins (53, 23%)

Fifty three (23%) of the salivary proteins identified in Table 1 were associated with the presence of a Chronic Inflammatory Response. Of the 53 proteins of this group, 10 (19%) were Th1/Th2 cytokines. The presence of VEGF, IL-10 and IL-6 may be a consequence of mutations in the serine/threonine-protein kinase B-Raf pathway (BRAF)^44^,^45^. Additionally, mutations in the BRAF oncogene also promotes the secretion of IL-1β, an innate inflammatory cytokine mediator which can drive neoplastic cells to up-regulate molecules that inhibit the function of anti-tumor lymphocytes. This may also be the reason for increased salivary presence of IL-1RA among cancer patients^44^,^45^.

Additionally, AKT1, CDKN1A, IFNγ, IL4, IL6, IL8, KRT14, KRT17, KRT5, SFN, STAT1 and p53 are associated with numerous overlapping pro-inflammatory, immunological pathways such as the glucocorticoid receptor regulatory network (GR) and the IFN-γ/Stat1 pathway^44^,^45^,^46^,^47^. The activated GR complex up-regulates the expression of anti-inflammatory proteins in the nucleus or represses the expression of pro-inflammatory proteins in the cytosol by preventing the translocation of other transcription factors from the cytosol into the nucleus. This coupled with the up-regulated activities of the IFN-γ/Stat1 pathway and the increase concentrations of TNF-α and IFN-γ may be significant as this suggests possible T cell exhaustion from constant exposure to tumor antigens or the absence of the HLA-A2 allele^44^,^45^,^46^,^47^.

### Cytoskeletal (40, 17%)

The cytoskeleton is present in all eukaryotic cells and provides the cell with structure, shape, mobility and by excluding macromolecules from some of the cytosol. It is also involved in cell migration and is believed to be involved in tumor dissemination^48^. Cytoskeleton protein complexes are also important regulators of migration, angiogenesis, cell polarity, cell morphology, intracellular trafficking and signal transduction. Many cytoskeleton proteins associated with cancer and are currently being used as histopathological biomarkers^48^,^49^,^50^.

The most abundant group in Table 1 is the cytokeratins. There are 16 cytokeratins among the cytoskeletal proteins. Nine of the cytokeratins are acidic Type 1 cytokeratins while seven are neutral Type II cytokeratins. The wide range of cytokeratin expression may result from the different types of salivary tissues contributing to the composition of whole saliva i.d., serous, mixed and mucinous secretions. However, similar to salivary gland ducts, normal breast ducts contain at least 3 types of epithelial cells: luminal (glandular) cells, basal/myoepithelial cells and stem cells^50^. Myoepithelial and luminal epithelia can be distinguished by their different cytokeratin expression patterns. Myoepithelial cells typically express cytokeratin 5/6 and cytokeratin 17, while luminal cells typically express cytokeratins 8 and 18. A small fraction of breast cancers express CK5 together with its major partners CK14 and CK17. Of particular interest is the presence of Cytokeratins 5, 14 and 17 as they are generally associated with poor prognosis and short disease-free survival^48^,^49^,^50^,^51^.

Within the salivary group of cytoskeletal proteins, of particular interest, are the proteins E-cadherin, γ-catenin, gesolin and vimentin which were down regulated in saliva. Together, these proteins are associated with the noncanonical planar cell polarity pathway which regulates the cytoskeleton that is responsible for the shape of the cell. E-cadherin to regulate b-catenin signaling in the canonical Wnt pathway; its potential to inhibit mitogenic signaling through growth factor receptors and the possible links between cadherins and the molecular determinants of epithelial polarity^52^,^53^. Each of these potential mechanisms provides insights into the complexity that is likely responsible for the tumor-suppressive action of E-cadherin^52^,^53^.

### Metabolic Proteins (56, 24%)

The metabolic proteins represent the largest of the nine categories of salivary proteins that were changed as a consequence of the presence of breast cancer. Table 1 shows that the 56 metabolic protein composed of 20 enzymes, 10 enzyme inhibitors, 10 transport proteins, 4 detoxification and redox proteins and 14 proteins of miscellaneous metabolic functions.

One interesting fact concerning the array of metabolic proteins is that alpha enolase, glucose-6-phosphate isomerase, fructose biphosphate aldolase, malate dehydrogenase, phosphoglycerate kinase 1, and triophosphate isomerase, are all associated with the glycolytic anaerobic pathway^54^,^55^,^56^,^57^,^58^,^59^. However, it is the presence of pyruvate kinase (PKM2) which suggests the occurrence of the Warburg effect^54^,^55^. The Warburg effect, suggested by Otto Warburg in 1927, suggests that among cancer cells there is a shift from ATP generation through oxidative phosphorylation to ATP generation through glycolysis even in the presence of normal oxygen concentrations^54^,^55^,^56^,^57^,^58^,^59^,^60^. The presence of the PKM2 protein suggests the occurrence of a glycolytic shift which is the metabolic hallmark of proliferating cells^54^,^55^,^56^,^57^,^58^,^59^,^60^,^61^,^62^.

A pathway analysis was performed on the list of these proteins and it revealed that ALDOA, ENOA, PGK1 and PKM2 are members of the Hypoxia-Inducible transcription Factor-1 alpha network (HIF-1α). The HIF-1α pathway deregulation can produce consequences in disease settings with a chronic inflammatory component. It has also been shown that chronic inflammation is self-perpetuating and that it distorts the microenvironment as a result of aberrantly active transcription factors. As a consequence, alterations in growth factor, chemokine, cytokine, and ROS balance occur within the cellular milieu that in turn provide the axis of growth and survival needed for *de novo* development of cancer and metastasis. It promotes angiogenesis and is consistent with the hypoxic events associated with the Warburg effect^54^,^55^,^56^,^57^,^58^,^59^,^60^,^61^,^62^,^63^.

### Membrane and Calcium Binding Related Proteins (18, 7%)

The most numerous proteins within this category are the annexins and the S100 family of proteins. The annexins are a large family of proteins which can be both intra and extracellular in presence having a wide variety of physiological functions^64^. They can be associated with membrane scaffolding, which is relevant to changes in the cell’s shape and have been shown to be involved in trafficking and organization of vesicles exocytosis. They are also associated with calcium ion channel formation. More importantly, annexins are associated with inflammation and apoptosis. With respect to carcinoma of the breast, the over expression of ANXA1, ANXA2, ANXA3 have been implicated with this malignancy^64^,^65^,^66^. ANXA1, expression, for example, was significantly *associated with* disease progression and metastases. ANXA2 and ANXA3 have also been related to poor prognosis and may have potential as prognostic indicators^67^,^68^.

S100 proteins, similar to the annexins, are implicated in a wide variety of intracellular and extracellular functions. For example, they are involved in regulation of protein phosphorylation, transcription factors, cytoskeleton dynamics, cell growth/differentiation, and the inflammatory response^69^,^70^,^71^,^72^.

A comprehensive study concerning the relationship between S100 proteins and breast cancer was conducted by Cancemi *et al*. in 2010^72^. The study identified the up-regulation of S100 protein expression in breast cancer tissues. Specifically, they observed S100A2, S100A4, S100A6, S100A7, S100A8, S10011 and S10013 as being disparate in the presence of breast cancer. Similarly, the proteins S100A4, S100A6, S100A7, S100A8 and S10011 were over expressed in saliva^72^.

### Anti-Microbial Proteins (14, 6%)

Table 1 illustrates 14 proteins that were not similar due to the presence of carcinoma of the breast. As illustrated, there are three mucins, which in health protect the integrity of the epithelia provide a barrier against microbial invasion. The balance of the panel consists of proline-rich proteins, lysozymes and histatins which have been documented in the dental literature as having antibacterial properties^73^,^74^.

The reason for their alteration in expression is unknown and may be due to changes in the oral microbiota^75^,^76^. As to whether the oral microbiome was altered as a consequence of tumor development or was an etiological cause for tumorigenesis to begin with, is also unknown. Our microbiota might be considered unknown variable at present, but they are likely to become more familiar considering the accelerated pace of research in this area^75^,^76^,^77^,^78^.

### Study Limitations

The manuscript presents a “proof of concept”, but has its limitations. Further study is required to address the phenotypic diversity breast cancer tumors. Tumors that are of different histological types, pathological grade and molecular subtypes should be proteomically assessed. Late stage tumors should also be studied and proteomically characterized according to the metastatic site of the tumor. High-throughput analysis is necessary in order to assay numerous individual specimens, where pooled specimens were used here to limit expense. The final task would be to combine the genomic, proteomic and other omic profiles together in an attempt to obtain a broader vision of how a carcinoma progresses.

## Conclusions

The authors of this manuscript have presented a catalogue of salivary proteins which are altered in concentration as a consequence of the presence of ductal carcinoma of the breast. These findings are supported by other investigators employing proteomic analysis of breast cancer cell lines, breast cancer tissues, tissue microenvironment and serum^79^,^80^,^81^. Additionally, nearly 29% of the panel of proteins has been technically validated by either western blot or by ELISA. A breakdown of these proteins has also been analyzed according staging and Her2/neu receptor status^12^,^82^. The investigators have also found that the protein concentrations can be modulated while undergoing cancer treatment and respond differently according pathological cell type^9^.

The main question arising from this line of research is how can tumors that are remote from the oral cavity influence salivary protein profiles? The prominent hypothesis to explain the aforementioned phenomenon is that exosomes which are shed and escape extracellular breakdown diffuse throughout the body and appear in biological fluids such as blood, urine, saliva semen breast milk, nipple aspirates and malignant effusions^83^. In doing so, they initiate exosome-mediated cell-to-cell communication by fusing with membranes of their cellular targets^84^,^85^. Upon entering the cellular he cytoplasm, their contents are released and activate downstream events in recipient cells^73^. It is also possible that extracellular proteases degrade the exosomes and their contents become soluble ligands binding to cell surface receptors. Experimentally, it has been demonstrated by Lau *et al*. that breast cancer-derived exosomes communicated and activated transcription within salivary gland cells and alter the proteomic composition of salivary gland cell-derived exosomes^86^.

Table 2 illustrates the presence of proteins carried by both salivary gland cell-derived exosomes and breast cancer-derived exosomes with 20 of the proteins being common to both cell types. The finding is circumstantial at this point in time as exosome research is in its infancy; however, as research continues in this field one could expect further support for the proposed mechanism for salivary protein alterations^73^,^74^,^83^,^84^,^85^.

In summary, the manuscript proposes a novel method for studying breast cancer progression which includes the tumor microenvironment and inflammatory progress^87^. Clinically, many of the markers have shown utility for monitoring treatment efficacy and tumor recurrence. Several proteins such as Lung Resistance Protein may be useful as a prognostic indicator. Additionally, key pathway markers such as EGFR, E-Cadherin, p53, Apaf-1, 14-3-3δ, Xiap, p21^WAF−1^ and others could be placed in a microarray assay and render a probability (risk assessment) of having carcinoma of the breast rather than a “yes or no” response which is subject to many false positive and negative assessments.

Further research is required in order determine the effects of tumor and tumor receptor phenotypes and the alteration of the panel as the tumor progresses from one stage to the next. It is also important to know which proteins from the profile are associated with tumor dormancy and tumor resistance to therapy. Considering that the salivary protein profile was modified secondary to the presence of a tumor remote from the oral cavity, it may provide information on the metastatic process which is the main cause of death for breast cancer patients.

## Methods and Materials

### Proteomic Design

The investigators analyzed pooled, stimulated whole saliva specimens. Each pooled specimen within a cohort consisted of ten individual patient saliva specimens from a bank of control and cancer specimens frozen at −80 °C. One pooled saliva specimen consisted of ten specimens from ten healthy volunteers, another specimen was a pooled saliva specimen from ten subjects diagnosed with ductal carcinoma *in situ* (DCIS)^10^. Similarly assembled pooled specimens came from Stage I, Stage IIa, Stage IIb, Her2/*neu* positive and Her2/negative ductal carcinoma volunteers^11^,^12^. The cancer cohort, internally, was estrogen and progesterone receptor status negative as determined by the pathology report. All subjects were closely matched for age and race and were non-tobacco users.

The study was conducted in accordance with the Declaration of Helsinki, and the University of Texas Health Science Center Institutional Review Board approved the protocol and informed consent form prior to study initiation. All participating volunteers were explained their participation rights and signed an IRB consent form. The saliva specimens and related patient data are non-linked and bar coded in order to protect patient confidentiality. This study was performed under the UTHSC IRB approved protocol# HSC-DB-05-0394.

To increase accuracy and assure reproducibility, a duplicate set of specimens were sent to the Harvard Partners Center for Genetics and Genomics, Cambridge, MA., and were proteomically analyzed using both bottom-up and gel based approaches. A Thermo Finnigan LTQ FT ICR Hybrid Mass Spectrometer was used for the proteome analysis.

In order to present a complete catalogue of salivary cancer related proteins, proteins from previous studies were added to this analysis. These proteins were determined by either antibody array and/or by ELISA^8^,^9^,^10^. The reason for adding these antibody based protein determinations was to provide information concerning the presence of some of the low abundance proteins that were changed secondary to carcinoma of the breast.

### Saliva Collection and Sample Preparation

Stimulated whole salivary gland secretion is a reflex response occurring during the mastication of a bolus of food. Usually, a standardized bolus (1 gram) of paraffin or a gum base (generously provided by the Wrigley Co., Peoria, IL) is given to the subject to chew at a regular rate. The individual, upon sufficient accumulation of saliva in the oral cavity, expectorates periodically into a preweighed disposable plastic cup between the hours of 8:00 a.m. and 5:00 p.m. As this is a reflexive collection, circadian rhythms are not a factor on salivary flow rates^10^. This procedure is continued for a period of five minutes. The volume and flow rate is then recorded along with a brief description of the specimen’s physical appearance^11^. Saliva specimens tainted with the presence of blood were not used in the study. The cup with the saliva specimen is reweighed and the flow rate determined gravimetrically. The specimens were placed on ice and immediately transported from the clinic to the laboratory for processing. Specimens were collected by one calibrated individual working in the same location.

The specimens were aliquoted and centrifuged in an Eppendorf centrifuge 5415R with temperature control (4 °C) for five minutes in order to remove debris and any unwanted particulates. The supernants were removed and a protease cocktail inhibitor (trypsin, calpain, papain, cathepsin B, chymotrypsin, kallikrein, human leukocyte elastase and aminopeptidases) from Sigma Co (St. Louis, MI, USA) was added along with enough dithiothreitol from a 1 M stock solution to bring its concentration 1 mM. The 1 ml aliquots were frozen at −80 °C^11^.

### Bottom-Up Mass Spectrometry Using iTRAQ Labeling

Briefly, the saliva samples were thawed and immediately centrifuged to remove insoluble materials^10^,^11^,^12^. The supernatants were assayed for protein using the Bio-Rad protein assay (Hercules, CA, USA) and an aliquot containing 100 μg of each specimen was precipitated with 6 volumes of −20 °C acetone. Specimens were normalized for analysis by using total protein concentrations. The precipitate was resuspended and treated according to the manufacturer’s instructions. Protein digestion and reaction with iTRAQ labels were carried out as previously described and according to the manufacturer’s instructions (Applied Biosystems, Foster City, CA). Briefly, the acetone precipitable proteins were centrifuged in a table top centrifuge at 15,000 × g for 20 minutes. The acetone supernatants were removed and their pellets resuspended in 20 ul dissolution buffer. The soluble fractions were denatured and the disulfides reduced by incubation in the presence of 0.1% SDS and 5 mM TCEP (tris-(2-carboxyethyl)phosphine)) at 60 °C for one hour. Cysteine residues were blocked by incubation at room temperature for 10 minutes with MMTS (methyl methane-thiosulfonate). Trypsin was added to the mixture to a protein: trypsin ratio of 10:1. The mixtures were incubated overnight at 37 °C. The protein digests were labeled by mixing with the appropriate iTRAQ reagent and incubating at room temperature for one hour. On completion of the labeling reaction, the four separate iTRAQ reaction mixtures were combined. Since there are a number of components that can interfere with the LC-MS/MS analysis, the labeled peptides were partially purified by a combination of strong cation exchange followed by reverse phase chromatography on preparative columns. The combined peptide mixtures were diluted 10 fold with loading buffer (10 mM KH_2_PO_4_ in 25% acetonitrile at pH 3.0) and applied by syringe to an ICAT Cartridge-Cation Exchange column (Applied Biosystems, Foster City, CA) column that has been equilibrated with the same buffer.

The column is washed with 1 ml loading buffer to remove contaminants. To improve the resolution of peptides during LC-MS/MS analysis, the peptide mixtures were partially purified by elution from the cation exchange column in 3 fractions. Stepwise elution from the column was achieved with sequential 0.5 ml aliquots of 10 mM KH_2_PO_4_ at pH 3.0 in 25% acetonitrile containing 116 mM, 233 mM and 350 mM KCl respectively. The fractions were evaporated Speed Vac to about 30% of their volume to remove the acetonitrile and then slowly applied to an Opti-Lynx Trap C18 100 ul reverse phase column (Alltech, Deerfield, IL) with a syringe. The column was washed with 1 ml of 2% acetonitrile in 0.1% formic acid and eluted in one fraction with 0.3 ml of 30% acetonitrile in 0.1% formic acid. The fractions were dried by lyophilization and resuspended in 10 ul 0.1% formic acid in 20% acetonitrile. Each of the three fractions was analyzed by reverse phase LC-MS/MS.

### Reverse Phase LC-MS/MS

The desalted and concentrated peptide mixtures were quantified and identified by nano-LC-MS/MS on an API QSTAR XL mass spectrometer (ABS Sciex Instruments) operating in positive ion mode. The chromatographic system consists of an UltiMate nano-HPLC and FAMOS autosampler (Dionex LC Packings). Peptides were loaded on a 75 μm × 10 cm, 3 μm fused silica C18 capillary column, followed by mobile phase elution: buffer (A) 0.1% formic acid in 2% acetonitrile/98% Milli-Q water and buffer (B): 0.1% formic acid in 98% acetonitrile/2% Milli-Q water. The peptides were eluted from 2% buffer B to 30% buffer B over 180 minutes at a flow rate 220 nL/min. The LC eluent was directed to a NanoES source for ESI/MS/MS analysis. Using information-dependent acquisition, peptides were selected for collision induced dissociation (CID) by alternating between an MS (1 sec) survey scan and MS/MS (3 sec) scans. The mass spectrometer automatically chooses the top two ions for fragmentation with a 60 s dynamic exclusion time. The IDA collision energies parameters were optimized based upon the charge state and mass value of the precursor ions.

Random control and cancer specimens from the specimen bank were selected and blindly sent for proteomic analysis to ascertain quantification repeatability and to address issues of variability, proteomic inconsistency and issues (pooled variance) surrounding the use of pooled specimens. Additionally, western blots were performed on both pooled and individual specimens for technical validation^88^,^89^,^90^,^91^.

### Bioinformatics and Statistical Methods

The accumulated LC-MS/MS spectra were analyzed by ProQuant and ProGroup software packages (Applied Biosystems) using the SwissProt database for protein identification. The ProQuant analyses were carried out with a 75% confidence cutoff with a mass deviation of 0.15 Da for the precursor and 0.1 Da for the fragment ions. The ProGroup reports were generated with a 95% confidence level for protein identification.

The Swiss-Prot database was employed for protein identification while the PathwayStudio^®^ bioinformatics software package was used to determine Venn diagrams were also constructed using the NIH software program (http://ncrr.pnl.gov). Pathways were retrieved from three databases: DAVID, KEGG, BioCarta, and the NCI’s Protein Interaction Database (PID)^86^,^92^,^93^,^94^,^95^. Gene ontologies were determined by employing the GO and AmiGO databases^95^,^96^.

Routine statistical evaluations were performed using the IBM SPSS Statistics 23 software. These evaluations include frequency, cross-tabulations and descriptive statistics. Mean comparisons were performed using parametric statistical analysis.

## Additional Information

**How to cite this article**: Streckfus, C. F. and Bigler, L. A Catalogue of Altered Salivary Proteins Secondary to Invasive Ductal Carcinoma: A Novel *In Vivo* Paradigm to Assess Breast Cancer Progression. *Sci. Rep*. **6**, 30800; doi: 10.1038/srep30800 (2016).

## Figures and Tables

**Table 1:** A Catalogue of Salivary Proteins Altered Secondary to Breast Cancer.

UniProt	Gene	Protein Name	Function	Stage
**Genomic Integrity Related Proteins (10, 4% of total)**				
P16403	H12	Histone H1.2	Transcription, DNA repair, replication	IIa, H^+^
**Q8IUE6**	**H2A2B**	**Histone H2A**	**Transcription, DNA repair, replication**	IIb
P16104	H2AX	Histone H2A.x (H2a/x)	Transcription, DNA repair, replication	IIa, H^+^
**Q99880**	**H2B1L**	**Histone H2B type 1-L**	**Transcription, DNA repair, replication**	IIb, H^+^
Q99877	H2B1N	Histone H2B type 1-N	Transcription, DNA repair, replication	0
Q16778	H2B2E	Histone H2B type 2-E	Transcription, DNA repair, replication	IIa, H^+^
**Q71DI3**	**H32**	**Histone H3.2**	**Transcription, DNA repair, replication**	IIa, H^+^
**P62805**	**H4**	**Histone H4**	**Transcription, DNA repair, replication**	IIa, H^+^
P12004	PCNA	Prolif. cell nuclear antigen	DNA repair	IIa, H^+^
**P35637**	**TLS**	**TLS Oncogene**	**Maintenance of genomic integrity**	IIa, H^+^
**Molecular Chaperones/Heat Shock Proteins (14, 6% of total)**				
**Q99933**	**BAG-1**	**BAG chaperone reg. 1**	**Inhibits chaperone activity HSP70/HSC70**	0, I, IIa, IIb, H^+^
Q6P5S2	CF058	C6orf58	Chromosome 6 open reading frame 58	IIa, H^+^
**P14211**	**CRP55**	**Calreticulin**	**Major endoplasmic reticular activity**	0, I, IIa, IIb, H^+^
P11021	GRP78	Glucose-regulated protein	Assembly multimeric prot. complexes	IIa, H^+^
P11142	HSP10	Heat Shock 10 protein	Repressor of transcriptional activation	IIb
Q12988	HSP27	Heat Shock 27 protein	Inhibitor of actin polymerization	I, IIa
P25685	Hsp40	Heat shock protein 40	Interacts with HSP70	IIa, H^+^
P08107	HSP70	Heat Shock 70 protein	Stabilizes preexistent proteins - aggregation	0, I, IIa, IIb, H^+^
**P20585**	**MSH3**	**DNA repair protein**	**Post-replicative DNA mismatch repair sys**	IIa, H^+^
P05307	PDI	Protein disulfide isomerase;	Catalyzes the rearrangement of -S-S- bonds	IIa, H^+^
**P07237**	**PDIA1**	**Protein disulfide-isomerase**	**Catalyzes rearrangement of -S-S- bonds**	IIb
**P62937**	**PPIA**	**Peptidyl-prolyl isomerase A**	**PPIases accelerate the folding of proteins**	IIa, IIb, H^+^
**P23284**	**PPIB**	**Peptidyl-prolyl isomerase B**	**PPIases accelerate the folding of proteins**	IIa, IIb, H^+^
**Q13546**	**RIP**	**Recept.-interacting protein 1**	**DNA damage repair**	IIa, H^+^
**Cell Growth Related Proteins (17, 7% of total)**				
**P63104**	**1433Z**	**14-3-3 protein zeta/delta**	**Protein kinase C inhibitor**	IIb
P31749	AKT-1	α- serine/threonine-prot. kinase	Cell survival, growth & angiogenesis	IIa, H^+^
P04040	CATA	Catalase	Promotes growth of cells	IIa, IIb
**P32577**	**Csk**	**Tyrosine-protein kinase CSK**	**Regulates cell growth**	IIa, H^+^
P01133	EGF	epidermal growth factor	Stimulates growth of epid. & epith. tissues	0, I, IIa, IIb, H^+^
P00533	EGFR	epidermal growth factor recep.	EGF receptor	0, I, IIa, IIb, H^+^
P09038	FGF	Fibroblastic growth factor	Growth factor	IIa, H^+^
P15692	VEGF	Vasc. Endothel. Growth Factor	Plays an important role in angiogenesis	IIa, H^+^
P29354	GRB2	Growth factor recept. protein 2	Links cell surface growth factor receptor	IIa, H^+^
P04626	HER2/neu	Epid. growth factor receptor 2	EGF receptor	0, I, IIa, IIb, H+
P15531	NDKA	Nucleoside diphosphate kin. A	Cell proliferation, differentiation	IIa, H^+^
**P29474**	**NOS3**	**Nitric oxide synthase**	**Mediates vascular angiogenesis**	IIa, H^+^
P29476	NOSI	NOS type I	Nitric oxide producer	IIa, H^+^
**P20936**	**Ras-GAP**	**Ras GTPase-act. prot. 1**	**Inhibitory reg. Ras-cyclic AMP pathway**	IIa, H^+^
P25815	S100P	S100-P	Stimulate cell proliferation	0
Q9UKW4	VAV3	VAV 3 oncogene	Plays an important role in angiogenesis	IIa, H^+^
P01135	TGF-α	Transforming growth factor α	Potent mitogenic polypeptide	0, I, IIa, IIb, H^+^
**Apoptosis Related Proteins (13, 5% of total)**				
**P31947**	**1433S**	**14-3-3 protein sigma**	**p53 regulated inhibitor**	IIa, IIb
**O14727**	**Apaf-1**	**Apop. prot. activity factor-1**	**Apoptosis activity**	IIa, H^+^
P99999	CYC	Cytochrome c	Apoptosis activity	IIa, H^+^
**P10909**	**CLU**	**Clusterin**	**Apoptosis activity**	0, I, IIa, H^+^
P80188	NGAL	Oncogene 24p3	Involved apoptosis, innate immunity	IIa, IIb
P38936	p21	WAF-1	Inhibitor of cellular proliferation	IIa, IIb, H^+^
P04637	p53	protein 53	Apoptosis	IIa, IIb, H+
**P19525**	**PKR**	**p68 kinase**	**Apoptosis, cell proliferation**	IIa, H^+^
P62988	UBIQ	Ubiquitin	Apoptosis	IIa, IIb, H^+^
P98170	XIAP	E3 ubiquitin-protein ligase XIAP	Apoptosis	IIa, H^+^
P04156	PRIP	Protein prion	Possible apoptotic activity	IIa
O43550	CDC25B	CDC25B	Tyrosine phosphatase activity	IIa, H^+^
P01375	TNF-α	Tumor necrosis factor alpha	Induces cell death.	0, I, IIa, IIb
**Immunity Related Proteins (52, 22% of total)**				
**P02763**	**A1AG1**	**Alpha-1 acid glycoprotein 1**	**Anti-inflammatory activity**	IIa, IIb, H^+^
P61769	B2MG	Beta-2 microglobulin precursor	Component of the MHC I complex	IIa,
**Q8N4F0**	**BPIL1**	**Palate, lung & nasal epith.**	**Involved in the innate immune response**	0, I, IIa, H^+^
**P01024**	**C3**	**Complement C3 precursor**	**Effector of innate and adaptive immunity**	0, I, IIa, IIb, H^+^
P0C0L4	C4	Complement 4A	Of the classical complement pathway	0, I, IIa, IIb, H^+^
**P28907**	**CD38**	**ADP-ribosyl cyclase 1**	**Receptor in cells of the immune system**	IIa, H^+^
P53618	COPB	Coatomer subunit beta	Degradation of CD4 & MHC I antigens	IIa
**P54108**	**CRIS3**	**Cysteine-rich secret. prot. 3**	**Ligand of alpha1B-glycoprotein in plasma**	0, IIa,
**Q9UGM3**	**DMBT1**	**GP 300**	**Interaction of tumor cells & immune system**	IIa, H^+^
**P08246**	**ELNE**	**Leukocyte elastase**	**Modifier of monocytes & granulocytes**	IIa, H^+^
P06241	FYN	Fyn-Tyrosine-protein kinase	Regulates cell growth	IIa, H^+^
P01762	HV301	Ig heavy chain V-III region TRO	Fc-epsilon receptor signaling pathway	IIb
**P01777**	**HV316**	**Ig heavy chain V-III region TEI**	**Fc-epsilon receptor signaling pathway**	IIa, H^+^
P01781	HV320	Ig heavy chain VIII region GAL	Complement activation	IIa, IIb
P01579	IFN-γ	Interferon gamma	Potent activator of macrophages	0, I, IIa, IIb, H^+^
P01876	IGHA1	Ig alpha1 chain C region	Defends against local infection	IIa, IIb
P01877	IGHA2	Ig alpha-2 chain C region	Defends against local infection	IIb, H^+^
**P01857**	**IGHG1**	**Ig gamma-1 chain C region**	**Complement activation**	0, I, IIa, IIb, H^+^
P01859	IGHG2	Ig gamma-2 chain C region	Complement activation	IIb, H^+^
P01591	IGJ	Immunoglobulin J chain	Links two monomer units of IgM or IgA	IIa, IIb
P05112	IL 4	Interleukin - 4	Activates B-cell and T-cell proliferation	0, I, IIa, IIb
P05231	IL 6	Interleukin - 6	Inducer of the acute phase response	0, I, IIa, IIb
P10145	IL 8	Interleukin - 8	A chemotatic factor	0, I, IIa, IIb
P22301	IL-10	Interleukin - 10	Cytokine Inhibitor (IFN-γ, IL-2, IL-3, TNF)	0, I, IIa, IIb
P18510	IL1RA	Interleukin-1 recept. antagonist	Antagonist to IL-1 alpha, beta	IIa, H^+^
Q9BY25	IL1β	IL-1beta	Inhibits neutrophil apoptosis	0, I, IIa, IIb
**P30740**	**ILEU**	**Leukocyte elastase inhibitor**	**Regulates neutrophil proteases**	IIb
P01834	KAC	Ig kappa chain C region	immunoglobulin κ chains	IIa, IIb, H^+^
P06870	KLK1	Kallikrein-1 precursor	Cleaves Met-Lys and Arg-Ser bonds	0, I, IIa, IIb
P06309	KV205	Ig kappa chain V-II region	Fc-epsilon receptor signaling pathway	IIa, H^+^
P18135	KV312	Ig kappa chain VIII region HAH	Surface immunoglobulin M autoantibody	IIa, IIb
P01842	LAC	Ig lambda chain C regions	Complement activation	IIa, H^+^
P06239	LCK	Lck-Tyrosine-protein kinase	Selection & maturation of T-cells in thymus	IIa, H^+^
P31025	LCN1	Lipocalin1 precursor	Regulates activity of neutrophil proteases	0, I, IIa, IIb, H^+^
P13500	MCP-1	Monocyte chemotactic prot.-1	Chemotactic factor attracts monocytes	0, I, IIa, IIb
O88888	Mint3	Minit-3	Activates macrophages	IIa, H^+^
P22894	MMP8	Matrix metalloproteinase-8	Degrades fibrillar type I, II & III collagens	IIa, H^+^
**P14780**	**MMP9**	**Matrix metalloproteinase-9**	**Basement membrane dissolution**	IIa, H^+^
**P01871**	**MUC**	**Ig mu chain C region**	**Role in primary defense mechanisms**	I, IIa, IIb, H^+^
**O43240**	**NES1**	**Kallikrein 10**	**Tumor suppressor in breast cancer**	IIa, H^+^
**P22079**	**PERL**	**Lactoperoxidase**	**Airway host defense against infection**	0, IIa, IIb, H^+^
**P05164**	**PERM**	**Myeloperoxidase**	**Host defense system of leukocytes**	IIa, IIb, H^+^
**P01833**	**PIGR**	**Poly-IG receptor protein**	**Binds IgA & IgM at basolateral surface**	IIa, IIb, H^+^
**P12273**	**PIP**	**Prolactin-inducible protein**	**Pathological conditions mammary gland**	I, IIa, IIb
P13796	PLSL	Plastin-2	Modulates cell surface expression IL2RA/CD25	IIa, H^+^
Q9NP55	PLUNC	BPI fold-containing family A1	Associated with tumor progression	0, I, IIa, IIb
P13501	RANTES	RANTES	Chemokine (CCL5)	0, I, IIa, IIb
P13405	Rb	Retinoblastoma-assoc. prot.	Acts as a tumor suppressor	IIa, H^+^
P29508	SPB3	Serpin B3	Immune response against tumor cells	IIa, H^+^
**P42224**	**Stat1**	**Transcription factor ISGF-3**	**Mediates responses to cytokines**	IIa, H^+^
P01135	TGF-α	Transforming growth factor α	Potent mitogenic polypeptide	0, I, IIa, IIb
P01375	TNF-α	Tumor necrosis factor alpha	Secreted to induce cell death	0, I, IIa, IIb
**Cytoskeleton Related Proteins (40, 17% of total)**				
P63261	ACTG	Actin, cytoplasmic 2	Cytoskeleton	IIa, H^+^
**Q01518**	**CAP1**	**Adenylyl cyclase**	**Actin cytoskeleton organization**	IIa, H^+^
**P12830**	**CDH1**	**Cadherin-1**	**Epithelial Adherens junction protein**	IIa, H^+^
P06731	CEA	Carcin-embryonic antigen	Cell adhesion and in intracellular signaling	0, I, IIa, IIb, H^+^
P23528	COF1	Cofilin, non-muscle isoform	Cytoskeleton	IIa, H^+^
**Q02487**	**DSC2**	**Desmocollin-2 precursor**	**Intercellular desmosome junctions**	IIb
P15311	EZRI	Ezrin	Cytoskeleton	0, I, IIa, IIb
**P02671**	**FIBA**	**Fibrinogen α chain precursor**	**Involved in cell adhesion, cell motility**	IIa,
P02675	FIBB	Fibrinogen beta chain precursor	Involved in cell adhesion, cell motility	0, I, IIa, IIb
P06396	GELS	Gelsolin	Assembly of monomers into filaments	IIa, H^+^
**Q15151**	**JUP**	**g-Catenin**	**Associated with desmosome junctions**	IIa, H^+^
**P13646**	**K1C13**	**Cytokeratin-13**	**Cytoskeleton protein**	0, IIa, IIb, H^+^
**P08779**	**K1C16**	**Cytokeratin-16**	**Cytoskeleton protein**	0, I, IIa, IIb
P35527	K1C9	Cytokeratin-9	Cytoskeleton protein	0, I, IIa, IIb, H^+^
**P04264**	**K2C1**	**Cytokeratin 1**	**Cytoskeleton protein**	0, I, IIa, IIb, H^+^
**P19013**	**K2C4**	**Cytokeratin 4**	**Cytoskeleton protein**	IIa, H^+^
**P13647**	**K2C5**	**Cytokeratin-5**	**Cytoskeleton protein**	0, I, IIa, IIb, H^+^
**P02538**	**K2C6A**	**Cytokeratin-6A**	**Cytoskeleton protein**	0, I, IIa, IIb, H^+^
P48666	K2C6C	Cytokeratin 6C	Cytoskeleton protein	0,
P13645	KRT10	Cytokeratin-10	Cytoskeleton protein	0, I, IIa, IIb, H^+^
P02533	KRT14	Cytokeratin-14	Cytoskeleton protein	0, I, IIb,
P19012	KRT15	Cytokeratin-15	Cytoskeleton protein	I, IIa,
Q04695	KRT17	Cytokeratin-17	Cytoskeleton protein	0, I, IIa, IIb, H^+^
P08727	KRT19	Cytokeratin-19	Cytoskeleton protein	IIa
P35908	KRT2	Cytokeratin 2	Cytoskeleton protein	0, I, IIa, IIb, H^+^
Q7Z3Z0	KRT25	Cytokeratin-25	Cytoskeleton protein	IIa, H^+^
P08729	KRT7	Cytokeratin-7	Cytoskeleton protein	0, I, IIa, IIb
P22894	MMP8	Matrix metalloproteinase-8	Degrades fibrillar type I, II & III collagens	IIa, H^+^
P14780	MMP9	Matrix metalloproteinase-9	Basement membrane dissolution	IIa, H^+^
P26038	MOES	Moesin	Involved in major cytoskeletal structures	IIa, H^+^
P49024	Paxillin	Paxillin	Involved in actin-membrane attachment	IIa, H^+^
**P07737**	**PROF1**	**Profilin-1**	**Binds to actin & affects the cytoskeleton**	0, IIa, IIb, H^+^
P03749	Rho	Rho	Regulates intracellular actin dynamics	IIa, H^+^
**P31949**	**S10AB**	**S100-A11**	**Cornification of keratinocytes**	IIa,
P35321	SPR1A	Cornifin-A	Keratinization activity	I, IIa, IIb, H^+^
P22528	SPR1B	Cornifin-B	Keratinization activity	IIb, H^+^
Q9UBC9	SPRR3	Cornifin beta	Envelope protein of keratinocytes	0, IIa, H^+^
P10636	TAU	Microtubule-associated prot. - τ	Promotes microtubule assembly and stability	IIa, H^+^
P62328	TYB4	Thymosin beta 4	Role in the organization of the cytoskeleton	IIa,
**P08670**	**VIME**	**Vimentin**	**Class-III intermediate filaments**	IIa, IIb, H^+^
**Metabolism Related Proteins (56, 24% of total)**				
P22303	ACTH	Acetylcholinesterase	Signal transduction at NMJ	IIa, H^+^
P07108	ACBP	AcylCoA binding protein	Binds medium & long-chain acyl-CoA	IIa, H^+^
Q15848	ADIPO	Adiponectin	Adipokine involved in fat metabolism	0, I, IIa, IIb, H^+^
Q9DCT1	AK1E1	Aldo-keto reductase	Catalyst	0,
**P04217**	**A1BG**	**Alpha-1B-glycoprotein**	**Functions as transport protein in blood**	IIa, H^+^
P01023	A2MG	Alpha-2 macroglobulin	Proteinase inhibitor	IIa, IIb, H^+^
Q92746	ST8SIA2	Alpha-2,8-sialyltransferase 8B	Protein modification; protein glycosylation	IIa, H^+^
**P19961**	**AMYC**	**Alpha-amylase 2B precursor**	**Carbohydrate metabolism**	0, I, IIa, IIb
**P06733**	**ENOA**	**Alpha-enolase**	**Enzyme that has a role in glycolysis**	IIa, IIb, H^+^
P03973	ALK1	Antileukoproteinase 1 precursor	Proteinase inhibitor	IIa, H^+^
**P02647**	**APOA1**	**Apolipoprotein A-I**	**Promotes efflux of cholesterol from cell**	IIb, H^+^
**P02649**	**APO-E**	**Apolipoprotein -E**	**Catabolizes lipoproteins**	IIa, H^+^
P06576	ATPB	ATP synthase subunit beta	ATP synthesis	0, IIa, IIb
Q8WZ76	BCRP	Breast cancer resistance prot.	ATP hydrolysis-dependent efflux transport	I
P00915	CAH1	Carbonic anhydrase 1	Reversible hydration of carbon dioxide	IIb,
**P23280**	**CAH6**	**Carbonic anhydrase 6**	**Reversible hydration of carbon dioxide**	0, I, IIa, IIb
P07339	CATD	Cathepsin D	Pathogenesis of breast cancer	0, I, IIa, IIb, H^+^
P01040	CYTA	Cystatin A	Protease inhibitor	0, I, IIa, H^+^
**P04080**	**CYTB**	**Cystatin B**	**Intracellular thiol proteinase inhibitor**	IIa, IIb, H^+^
**P01034**	**CYTC**	**Cystatin C**	**Local regulator of this enzyme activity**	0, I, IIa, IIb, H^+^
**P28325**	**CYTD**	**Cystatin-D**	**Proteinase inhibitor**	0, I, IIa, IIb, H^+^
**P01036**	**CYTS**	**Cystatin-S precursor**	**Protein inhibits papain and ficin**	0, I, IIa, IIb
**P09228**	**CYTT**	**Cystatin-SA precursor**	**Thiol protease inhibitor**	IIa, IIb, H^+^
**P01037**	**CYTN**	**Cystatin-SN precursor**	**Cysteine proteinase inhibitors**	IIa, IIb
P20813	CYP2B6	Cytochrome p450	NADPH-dependent electron transport	0, I, IIa, IIb, H^+^
Q01469	FABPE	Fatty acid binding protein	Involved in keratinocyte differentiation	IIa, H^+^
**P80303**	**NUCB2**	**Gastric cancer antigen Zg4**	**Calcium-binding protein**	0, IIa, IIb, H^+^
**P06744**	**G6PI**	**Glucose-6-phosphate isom.**	**Glycolytic enzyme**	IIb
**P09211**	**GSTP1**	**Glutathione S-transferase P**	**Regulates negatively CDK5 activity**	IIa, IIb, H^+^
**P00738**	**HPT**	**Haptoglobin precursor**	**Captures & combines with hemoglobin**	0, IIa, IIb, H^+^
Q9Y5Z4	HEBP2	Heme-binding protein 2	Promotes mitochondrial permeability transition	IIa
P69905	HBA	Hemoglobin subunit alpha	Oxygen transport from lung	IIb, H^+^
P68871	HBB	Hemoglobin subunit beta	Oxygen transport from lung	IIb, H^+^
P02790	HEMO	Hemopexin precursor	Binds heme and transports to liver	IIb
**P04075**	**ALDOA**	**Fructose biphosphate aldol.**	**Role in glycolysis & gluconeogenesis**	IIa, IIb
Q14764	LRP	Lung resistance-related protein	Role in nucleo-cytoplasmic transport	I
P40926	MDHM	Malate dehydrogenase	Catalytic activity	0, IIa, IIb, H^+^
O15438	MRDP	Multidrug resistance protein	Inducible transporter of organic anions	I
**Q06830**	**PRDX1**	**Peroxiredoxin-1**	**Redox regulation of the cell**	IIa, IIb, H^+^
P30041	PRDX6	Peroxiredoxin-6	Redox regulation of the cell	IIa,
P00558	PGK1	Phosphoglycerate kinase 1	Glycolytic enzyme	IIa, IIb, H^+^
Q96PX9	PKH4B	Pleckstrin family G member 4B	PH domains bind various proteins	IIa,
P20742	PZP	Pregnancy zone protein	Inhibits all four classes of proteinases	0, I, IIa, IIb, H^+^
**P08129**	**PP1**	**Protein phosphatase 1**	**Regulation of glycogen metabolism**	IIa, H^+^
**Q08188**	**TGM3**	**Transglutaminase E**	**Formation of isopeptide cross-links**	IIa, H^+^
P14618	PKM2	Pyruvate kinase PKM2	Caspase independent cell death of tumor cells	IIa, IIb, H^+^
**P02768**	**ALBU**	**Serum albumin precursor**	**Regulation of colloidal osmotic pressure**	0, I, IIa, IIb
**P10599**	**THIO**	**Thioredoxin**	**Cytoplasmic antioxidant**	IIb
**P37837**	**TALDO**	**Transaldolase**	**Balances metabolites**	IIb, H^+^
P20061	TCO1	Transcobalamin1 precursor	Vitamin B12-binding protein	IIa, IIb
**P02787**	**TRFE**	**Transferrin**	**Iron transporter**	0, IIa, IIb, H^+^
**P29401**	**TKT**	**Transketolase**	**Transfers ketol group to aldose acceptor**	IIa,
P60174	TPIS	Triosephosphate isomerase	Catalytic activity	IIa, H^+^
P02774	VTDB	Vitamin D-binding protein	Transport	IIb
P25311	ZA2G	Zinc-alpha-2-glycoprotein	Stimulates lipid degradation in adipocytes	0,
Q96DA0	U773	Zymogen granule protein	Protein trafficking	IIa, H^+^
**Membrane and Calcium Binding Related Proteins (17, 7% of total)**				
**P04083**	**ANXA1**	**Annexin A1**	**Membrane fusion & exocytosis**	0, I, IIa, IIb, H^+^
**P46193**	**ANXA1**	**Annexin I**	**Membrane fusion & exocytosis**	0, I, IIa, IIb, H^+^
**P07355**	**ANXA2**	**Annexin II**	**Calcium-regulated membrane-binding prot.**	0, I, IIa, IIb, H^+^
P12429	ANXA3	Annexin A3	Inhibitor of phospholipase A2	IIb,
P02765	FETUA	Alpha-2-Z-globulin	Promotes endocytosis	IIa, IIb
P52566	GDIS	Rho GDP-dissociation inhib. 2	Regulates GDP/GTP exchange reaction	IIa, IIb
P20810	ICAL	Calpastatin	Specific inhibition of calpain	IIa,
P97799	p24	Neurensin-1	Role in neural organelle transport	IIa, H^+^
P15154	Rac1	p21 Rac-1	Plasma membrane-associated small GTPase	IIa, H^+^
**P11233**	**Ral A**	**Ras-related protein Ral-A**	**GTPase involved in cellular processes**	IIa, H^+^
P62158	CALM	Calmodulin	Control of a large number of enzymes	IIb
P26447	S10A4	S100-A4	Regulation of I-kappaB kinase/NF-kappaB	IIa,
**P06703**	**S10A6**	**S100-A6**	**Modulator of cellular calcium signaling**	IIa,
P31151	S100P	S100-A7	Calcium binding protein	0, I, IIa, IIb, H^+^
**P05109**	**S10A8**	**S100-A8**	**Inflammatory processes, immune response**	0, I, IIa, IIb, H^+^
**P06702**	**S10A9**	**S100-A9**	**Inflammatory processes, immune response**	0, I, IIa
P80511	S10AC	S100-A12	Binding protein, immune response	0, I, IIa, IIb, H^+^
**Oral Anti-Microbial Related Proteins (14, 6% of total)**				
P08311	CATG	Cathepsin G	Antibacterial to Gram-negative bact.	0, IIb
P59666	DEF3	Neutrophil defensin 3	Antimicrobial to Gram-negative/positive bact.	I, IIa, IIb
**P15515**	**HIS1**	**Histatin-1 precursor**	**Exhibit antibacterial & antifungal activities**	IIa, IIb
**P61626**	**LYSC**	**Lysozyme C precursor**	**Bacteriolytic function**	IIa, IIb
**Q9HC84**	**MUC5B**	**Mucin-5B precursor**	**Clearance of bacteria in the oral cavity**	0, I, IIa, IIb, H^+^
Q8TAX7	MUC7	Mucin-7	Clearance of bacteria in the oral cavity	0, I, IIa, H^+^
**P02812**	**PRB2**	**Salivary prol-rich prot. 2**	**Oral antimicrobial activity**	IIa, H^+^
Q16378	PROL4	Proline-rich protein 4	Antimicrobial activity	IIa, H^+^
P02814	SMR3B	Submax. gland androgen-reg. 3	Salivary PRP	0, I, IIa, H^+^
**Q96DR5**	**SPLC2**	**Parotid secretory protein**	**Innate immune response**	0, I, IIa, IIb, H^+^
**P02788**	**TRFL**	**Lactotransferrin**	**Antimicrobial activity**	0, IIa, IIb, H^+^
**P02810**	**PRPC**	**Acidic proline-rich prot.**	**Inhibitors of crystal growth**	IIa, IIb
P15941	MUC-1	Cancer antigen 15-3	Adhesion and an anti-adhesion protein	0, I, IIa, IIb, H^+^
Q02817	MUC-2	Mucin 2	Mucosal protection	IIa, IIb

Note: The UniProt identification number, gene identification protein name and protein function are from UniProt database (www.uniprot.org). The proteins are classified according to molecular function. Additionally all down-regulated proteins are bold; otherwise, the remaining proteins are up-regulated.

Staging Abbreviations: 0 = Stage 0, I = Stage I, IIa = Stage IIa, IIb = Stage IIb, H^+^ = Her2/neu positive.

**Table 2:** Salivary Protein Presence in Breast Cancer Cell Lines and Exosomes.

UniProt	Protein Name	Method of Identification	Presence in Cell Lines	Exosomes In Saliva	Exosomes in T.T.	Ref.
**Genomic Integrity Related Proteins**						
P16403	Histone H1.2	MS	TT			25
Q8IUE6	Histone H2A	MS	TT			25
Q99880	Histone H2B type 1-L (H2B.c)	MS	TT			25
Q71DI3	Histone H3.2	MS	TT			28
P62805	Histone H4	MS		Yes		73
P12004	Prolif. cell nuclear antigen	AA	T, TT			8, 20, 77
**Molecular Chaperones/Heat Shock Proteins**						
Q6P5S2	C6orf58	2D, MS		Yes		85
P27797	Calreticulin	MS, AA	S, B, M, MD, TT		Yes	8, 15, 16, 17,
P11021	Glucose-regulated protein	AA	S, B, M, MD, TT	Yes	Yes	8, 15, 16, 17, 74, 85
P11142	Heat Shock 10 protein	MS		Yes	Yes	74, 85
Q12988	Heat Shock 27 protein	MS	B, M, MD, TT			14, 15, 16
P08107	Heat Shock 70 protein	MS	B, M, T	Yes	Yes	15, 16, 74, 85
P05307	Protein disulfide isomerase; p55	AA		Yes		8, 15, 16, 17, 85
P07237	Protein disulfide-isomerase	MS	B, M, MD, TT			14, 15, 16
P62937	Peptidyl-prolyl cis-trans isom. A	MS	S, B, M, MD	Yes	Yes	15, 16, 17, 74, 85
**Cell Growth Related Proteins**						
P63104	14-3-3 protein zeta/delta	AA	B, M, MD, TT	Yes		8, 15, 18, 85
P00533	epidermal growth factor receptor	E	S, M	Yes	Yes	10, 17, 74, 85
P29354	Growth factor receptor protein 2	AA	S, M, MD	Yes		8, 17, 85
P04626	epidermal growth factor receptor2	WB, E	S	Yes	Yes	10, 17, 74, 85
P15531	Metastatic process-associ. prot.	AA	M			8
**Apoptosis Related Proteins**						
P31947	14-3-3 protein sigma	MS	TT			18, 37
P38936	WAF-1	AA, E	S			8, 17, 39
P04637	protein 53	E	TT			8, 36
P62988	Ubiquitin	MS, WB	S, B	Yes	Yes	15, 16, 17, 85
**Immunity Related Proteins**						
P61769	Beta-2 microglobulin precursor	MS	S, B	Yes	Yes	15, 16, 17, 74, 85
Q8N4F0	Long palate, lung and nasal epith.	MS		Yes	Yes	74, 85
Q9UGM3	GP 300	MS		Yes		85
P01579	Interferon gamma	AA	TT			8, 57
P01876	Ig alpha1 chain C region	MS		Yes		85
P01877	Ig alpha-2 chain C region	MS		Yes		85
P01857	Ig gamma-1 chain C region	MS		Yes		15, 85
P01859	Ig gamma-2 chain C region	MS		Yes		85
P01591	Immunoglobulin J chain	MS		Yes		85
P05112	Interleukin - 4	AA, E	TT			8, 10, 87
P05231	Interleukin - 6	AA	TT			8, 46, 87
P10145	Interleukin - 8	AA	TT			8, 46, 87
P22301	Interleukin - 10	AA	M, TT			8, 46, 87
Q9BY25	IL-1beta	AA	TT			8, 46, 87
P30740	Leukocyte elastase inhibitor	MS	M, MD			85
P01834	Ig kappa chain C region	MS		Yes		85
P01842	Ig lambda chain C regions	MS		Yes		85
P31025	Lipocalin1 precursor	MS	TT			85
P01871	Ig mu chain C region	MS		Yes		85
P22079	Lactoperoxidase	MS		Yes		85
P05164	Myeloperoxidase	MS		Yes		85
P01833	Poly-IG receptor protein	MS	TT			85
P12273	Prolactin-inducible protein	MS		Yes		85
P26447	S100-A4	MS	S			17
P05109	S100-A8	MS, WB	S, TT	Yes		18, 85
P06702	S100-A9	MS	S	Yes		17
P29508	Serpin B3	MS	M, MD	Yes		14, 85
P42224	STAT-1	MS, AA	S, M			17, 80
P01135	Transforming growth factor alpha	AA	S			8, 17
P01375	Tumor necrosis factor alpha	AA	TT			8, 17
**Cytoskeleton Related Proteins**						
P63151	Actin, cytoplasmic 2	MS	S, B			15, 16, 17
Q01518	Adenylyl cyclase	MS	S			17
P12830	Cadherin-1	MS, AA	MD, TT			8, 15, 16, 17
P06731	Carcin-embryonic antigen	MS		Yes	Yes	85
P23528	Cofilin, non-muscle isoform	MS	S, B, M, MD, TT		Yes	14, 18
P15311	Ezrin	MS	S, B	Yes	Yes	17, 80, 85
P06396	Gelsolin	MS	S	Yes	Yes	17, 18, 85
P13646	Cytokeratin-13	MS	S			17
P08779	Cytokeratin-16	MS		Yes		85
P35527	Cytokeratin-9	MS	S, B		Yes	15
P35908	Keratin, type II	MS	S			17
P04264	Cytokeratin 1	MS	S, M, MD	Yes	Yes	17
P19013	Cytokeratin 4	MS		Yes		85
P13647	Cytokeratin-5	MS	S			28
P02538	Cytokeratin-6A	MS		Yes		80
P13645	Cytokeratin-10	MS	S			17, 87
P02533	Cytokeratin-14	MS	TT	Yes		85
P19012	Cytokeratin-15	MS	M, MD, TT	Yes		85
Q04695	Cytokeratin-17	MS	TT			45
P08727	Cytokeratin-19	MS	S, TT	Yes		17, 87
P08729	Cytokeratin-7	MS	S, TT			17, 80, 85
P26038	Moesin	MS	M, MD	Yes	Yes	80
P07737	Profilin-1	MS, WB, E	S, B, M, MD, T	Yes	Yes	15, 16, 17, 18, 19, 20, 21, 22, 23, 24, 25, 26, 27, 85
P03749	Rho	AA	S			8, 15, 17
P31949	S100-A11	MS	TT	Yes	Yes	85
P35321	Cornifin-A	MS		Yes	Yes	85
P08670	Vimentin	MS	S, B, M, MD		Yes	15, 16, 17
**Metabolism Related Proteins**						
P07108	AcylCoA binding protein	AA	B		Yes	15, 16
Q9DCT1	Aldo-keto reductase	MS	TT			14
P06733	Alpha-enolase	MS, WB, E	S, B, M, MD, TT	Yes	Yes	14, 15, 16, 17, 18, 85
P03973	Antileukoproteinase 1 precursor	MS		Yes		85
P02647	Apolipoprotein A-I	MS	S, TT	Yes	Yes	15, 17
P02649	Apolipoprotein -E	AA		Yes		8, 15, 85
P06576	ATP synthase subunit beta	MS	M, MD			80
P00915	Carbonic anhydrase 1	MS	TT		Yes	14, 18
P23280	Carbonic anhydrase 6	MS		Yes		85
P07339	Cathepsin D	MS, AA, E	M, MD, T, S			8, 14, 17
P04080	Cystatin B	MS	S	Yes		17, 85
P01034	Cystatin C	MS	B			16
P28325	Cystatin-D	MS		Yes		85
P01036	Cystatin-S precursor	MS		Yes		85
P09228	Cystatin-SA precursor	MS		Yes		85
P01037	Cystatin-SN precursor	MS		Yes		85
Q01469	Fatty acid binding protein	MS	S, TT	Yes	Yes	8, 18, 85
P06744	Glucose-6-phosphate isomerase	MS	S, M, MD		Yes	14, 17
P09211	Glutathione S-transferase P	MS	S, B, M, MD, TT			14, 15, 16, 17, 18
P04075	Fructose biphosphate aldolase	MS	M, MD			14
P40926	Malate dehydrogenase	MS	S, M, MD, T			17
Q06830	Peroxiredoxin-1	MS	S, B, TT	Yes		15, 16, 17, 85
P30041	Peroxiredoxin-6	MS	B, M, MD, TT			14, 15, 16,
P00558	Phosphoglycerate kinase 1	MS	S	Yes	Yes	17, 85
P14618	Pyruvate kinase PKM2	AA	B, M, MD	Yes		14, 15, 16, 18, 85
P10599	Thioredoxin	MS	S, B, TT	Yes	Yes	15, 16, 17, 85
P37837	Transaldolase	MS	S			17
P29401	TranSetolase	MS	S			17
P60174	Triosephosphate isomerase	MS	B, M		Yes	14, 15, 16
P25311	Zinc-alpha-2-glycoprotein	MS, 2DMS, E	S, MC	Yes		17
Q96DA0	Zymogen granule protein	MS		Yes		85
**Membrane Related Proteins**						
P04083	Annexin A1	AA	S, B, M, MD, TT	Yes	Yes	15, 16, 17, 74, 85
P46193	Annexin I	MS, WB	S, B, M, MD			15, 16, 17
P07355	Annexin II	MS, WB	S, B, M, MD, TT	Yes	Yes	14, 18, 74, 85
P12429	Annexin A3	MS		Yes		14, 85
P15154	p21 Rac-1	AA	S			8, 17
P11233	Ras-related protein Ral-A	AA	TT			8, 18
**Calcium Binding Related Proteins**						
P62158	Calmodulin	MS	B, M, MD	Yes	Yes	15, 16, 74, 85
P26447	S100-A4	MS	S, B			15, 16, 17
P06703	S100-A6	MS	S	Yes	Yes	17, 74, 85
P31151	S100-A7	MS	S, TT			16, 17
**Anti-Microbial Related Proteins**						
P59666	Neutrophil defensin 3	MS		Yes		85, 87
P15515	Histatin-1 precursor	MS		Yes		85
Q9HC84	Mucin-5B precursor	MS		Yes		85
Q8TAX7	Mucin-7	MS		Yes		85
P02814	Submax. gland androgen-reg. prot.	MS, 2DMS, E	S, M, MB			17
P02788	Lactotransferrin	MS		Yes		85
P15941	Cancer antigen 15-3	E		Yes	Yes	29, 85

Abbreviations: The UniProt identification number and protein name are from UniProt database (www.uniprot.org). Method of Identification: MS = Mass spectrometry, 2DMS = 2D-Gel Spot Mass Spectrometry, E = ELISA, WB = Western blot, AA = Antibody Array. Cell Lines: S = SKBR3, M = MCF7, T = T47D, MD = MB-MDA-231, B = 8701-BC, TT = Tumor Tissue. Ref. = References.

**Table 3:** Altered Salivary Proteins According to Molecular Function.

Go Function	Go ID	Gene Frequency & %
Anatomical Structure Development	GO:0048856	94 of 204 genes, 46%
Biosynthetic Process	GO:0009058	63 of 204 genes, 46%
Carbohydrate Metabolic Process	GO:0005975	21 of 204 genes, 10%
Catabolic Process	GO:0009056	54 of 204 genes, 46%
Cell Adhesion	GO:0007155	26 of 204 genes, 13%
Cell Cycle	GO:0007049	22 of 204 genes, 11%
Cell to Cell Signaling	GO:0007267	19 of 204 genes, 9%
Cell Death	GO:0008219	65 of 204 genes, 46%
Cell Differentiation	GO:0030154	62 of 204 genes, 46%
Cell Proliferation	GO:0008283	50 of 204 genes, 25%
Cellular Component Assembly	GO:0022607	45 of 204 genes, 22%
Cellular Nitrogen Metabolic Process	GO:0034641	73 of 204 genes, 46%
Cellular Protein Modification Process	GO:0006950	53 of 204 genes, 46%
Chromosome Organization	GO:0051276	14 of 204 genes, 7%
Cytoskeleton	GO:0007010	25 of 204 genes, 12%
DNA Metabolic Process	GO:0006259	27 of 204 genes, 13%
Generation of metabolites & Energy	GO:0006091	14 of 204 genes, 7%
Growth	GO:0040007	26 of 204 genes, 13%
Homeostatic Process	GO:0042592	53 of 204 genes, 46%
Immune System	GO:0002376	83 of 204 genes, 42%
Lipid Metabolic Process	GO:0006629	24 of 204 genes, 12%
Membrane Organization	GO:0061024	18 of 204 genes, 9%
Morphogenesis	GO:0006950	25 of 204 genes, 12%
Response to Stress	GO:0006950	107 of 204 genes, 52%
Signal Transduction	GO:0007165	86 of 204 genes, 46%
Small Molecule Metabolic Process	GO:0044281	50 of 204 genes, 25%
Transmembrane Transport	GO:0055085	11 of 204 genes, 5%
Transport	GO:0006810	89 of 204 genes, 52%
Vesicle-mediated Transport	GO:0016192	49 of 204 genes, 24%

**Table 4:** National Cancer Institute’s Pathway Interaction Database Analysis.

Pathway	Proteins	p value
Signaling events mediated by HDAC Class III	CDKN1A, HIST1H4A, HIST1H4B, HIST1H4C, HIST1H4D, HIST1H4E, HIST1H4F, HIST1H4H, HIST1H4I, HIST1H4J, HIST1H4K, HIST1H4L, HIST2H4A, HIST2H4B, HIST4H4, TP53	6.22E-18
Glucocorticoid receptor regulatory network	AKT1, CDKN1A, IFNG, IL4, IL6, IL8, KRT14, KRT17, KRT5, SFN, STAT1, TP53	6.73E-08
VEGFR1 specific signals	AKT1, CALM1, CALM2, CALM3, NOS3, RASA1, VEGFA	1.52E-06
AP-1 transcription factor network	BAG1, CCL2, IFNG, IL10, IL4, IL6, IL8, MMP9, TP53	9.53E-06
Caspase Cascade in Apoptosis	AKT1, APAF1, ARHGDIB, GSN, RIPK1, TNF, VIM, XIAP	1.43E-05
Insulin-mediated glucose transport	AKT1, CALM1, CALM2, CALM3, SFN, YWHAZ	1.84E-05
SHP2 signaling	EGF, EGFR, IFNG, IL6, LCK, NOS3, STAT1, VEGFA	1.85E-05
a6b1 and a6b4 Integrin signaling	AKT1, CDH1, EGF, EGFR, ERBB2, SFN, YWHAZ	2.94E-05
Angiopoietin receptor Tie2-mediated signaling	AKT1, CDKN1A, FGF2, FYN, NOS3, RASA1, TNF	4.45E-05
ErbB1 downstream signaling	AKT1, CALM1, CALM2, CALM3, EGF, EGFR, RALA, SFN, STAT1, YWHAZ	5.51E-05
IFN-gamma pathway	AKT1, CALM1, CALM2, CALM3, IFNG, STAT1	1.80E-04
ErbB receptor signaling network	EGF, EGFR, ERBB2, TGFA	2.31E-04
HIF-1-alpha transcription factor network	AKT1, ALDOA, ENO1, PGK1, PKM, TF, VEGFA	3.18E-04
Calcium signaling in the CD4+ TCR pathway	CALM1, CALM2, CALM3, IFNG, IL4	3.20E-04
LKB1 signaling events	CTSD, EZR, MAPT, SFN, TP53, YWHAZ	3.29E-04
Ceramide signaling pathway	AKT1, CTSD, EGF, EIF2AK2, RIPK1, TNF	3.68E-04
Direct p53 effectors	APAF1, CDKN1A, CTSD, EGFR, HSPA1A, HSPA1B, PCNA, SFN, TGFA, TP53	3.82E-04
Signaling events mediated by PTP1B	AKT1, EGF, EGFR, FYN, LCK, TXN	5.05E-04
Signaling events mediated by VEGFR1 and VEGFR2	AKT1, CALM1, CALM2, CALM3, FYN, NOS3, VEGFA	5.30E-04
EGF receptor (ErbB1) signaling pathway	EGF, EGFR, GSN, RASA1, STAT1	5.66E-04
